# Divergent Effect of Tacalcitol (PRI-2191) on Th17 Cells in 4T1 Tumor Bearing Young and Old Ovariectomized Mice

**DOI:** 10.14336/AD.2019.0618

**Published:** 2020-03-09

**Authors:** Agata Pawlik, Artur Anisiewicz, Beata Filip-Psurska, Dagmara Klopotowska, Magdalena Maciejewska, Andrzej Mazur, Joanna Wietrzyk

**Affiliations:** ^1^Department of Experimental Oncology, Hirszfeld Institute of Immunology and Experimental Therapy, Polish Academy of Sciences, Wroclaw, Poland; ^2^Université Clermont Auvergne, INRA, UNH, Unité de Nutrition Humaine, F-63000 Clermont-Ferrand, France

**Keywords:** calcitriol, PRI-2191, Th17, IL-17A, breast cancer, metastasis

## Abstract

Vitamin D and its analogs are known for their role in the development of breast cancer and in immunomodulation. Our previous studies have shown the pro-metastatic effect of calcitriol and tacalcitol (PRI-2191) in young mice bearing 4T1 breast cancer and the anti-metastatic effect in aged ovariectomized (OVX) mice. Therefore, the aim of our work was to characterize Th17 cell population in young and aged OVX mice bearing 4T1 tumors treated with calcitriol and PRI-2191. The expression of genes typical for Th17 cells was examined in splenocytes, as well as splenocytes differentiated with IL-6 and TGF-β to Th17 cells (iTh17). Expression of genes encoding vitamin D receptor (*Vdr*) and osteopontin (*Spp1*) as well as the secretion of IL-17A were evaluated in iTh17 cells. PRI-2191 treatment increased the expression of *Rora* and *Rorc* transcription factors, *Il17a*, *Il17re* and *Il21* in iTh17 cells from young mice. In aged OVX mice this effect was not observed. Increased expression was observed in the case of *Vdr* and *Spp1* genes in iTh17 cells from young mice treated with PRI-2191. What is more, in young mice treated with PRI-2191 the secretion of IL-17A to the culture media by iTh17 cells was increased, whereas in aged OVX mice a significant decrease was noted. Increased expression of *Spp1* in young mice treated with PRI-2191 may enhance the differentiation of Th17 cells.

Patients suffering from metastatic disease or those who develop metastases after successful treatment of the primary tumor have generally poor prognosis. During the complex and multistage process of metastasis, the tumor cells must evade an attack of the immune system [1]. However, continued tumor-conducive inflammation, widely recognized as potentiating primary tumor development, is also considered as favorable to the neoplastic metastasis [2,3]. It includes the infiltration of leukocytes, the presence of polypeptide messengers of inflammation, and the occurrence of tissue remodeling and angiogenesis. Inflammatory cells and cytokines found in tumors are more likely to contribute to tumor growth, progression, and immunosuppression rather than to enhance the host’s effective anti-tumor response. While some leukocytes i.e. cytotoxic T lymphocytes and natural killer (NK) cells certainly have this potential, other leukocyte cell populations, mainly innate immune cells i.e. mast cells, immature myeloid cells, granulocytes, and macrophages potentiate tumor progression and enhance cancer cell survival [4,5]. It is known that the tumor cells and tumor-associated fibroblasts build an inflammatory milieu favorable for Th17 cells recruitment [6]. In breast cancer Th17 cells infiltration is a poor prognosis factor [7]. Moreover, infiltration with Th17 cells or IL-17A producing cells is preferably observed in estrogen receptor negative (ER-), progesterone receptor negative (PR-) and triple negative tumors [8,9]. IL-17 produced mainly by Th17 lymphocytes is an important cytokine in tumor progression, angiogenesis and metastasis [10]. Cochaud et al. also proved, that recombinant IL-17A induces the MAPK pathway by upregulating phosphorylated ERK1/2 in human breast cancer cell lines, thereby promoting proliferation and resistance to conventional chemotherapeutic agents such as docetaxel and stimulates migration and invasion of breast cancer cells [9]. However, this cytokine, as well as Th17 cells (probably due to the high plasticity) are described as driving pro- or anti-tumor response [10,11]. An example of animal studies showing pro-tumoral and pro-angiogenic activity of IL-17 are studies by Du et al. utilizing triple-negative 4T1 mouse mammary gland cancer model [12]. The 4T1 orthotopic tumor growth is associated with high immune response including large leukocytosis, lung and tumor neutrophil infiltration etc. [13]. In their work, Du et al. have shown that injection of recombinant IL-17 promotes the growth and microvascular density of 4T1 tumors [12]. Recently, in the same tumor model we have shown that the active form of vitamin D_3_ (calcitriol) and its two low calcemic analogs: PRI-2191 and PRI-2205 increased metastatic spread when tumors were growing in young mice [14]. However, in aged, ovariectomized (OVX) mice, a transient anti-metastatic effect was observed [15].

Immune cells express vitamin D receptor (VDR) and are capable to metabolize vitamin D. VDR is constitutively expressed by dendritic cells and macrophages. Its expression is also induced in lymphocytes following activation, indicating an important role for vitamin D in the modulation of immune and inflammatory responses [16]. In general, vitamin D is considered an anti-inflammatory molecule, which would be beneficial for cancer prevention and therapy (references in recently published review: [17]). However, other authors have suggested that such properties of vitamin D may lead to unfavorable effects in cancer treatment [16].

Vitamin D deficiency and the low tumor expression level of VDR are correlated with aggressive breast cancer characteristic [18-20]. Also preliminary analysis of breast cancer circulating tumor cells suggests VDR as a potential prognostic biomarker [21]. The data showing promotion of tumor growth and metastasis via vitamin D deficiency are presented also using xenografts of human breast cancer cell lines [22] and allografted mouse tumors [23-25]. The anticancer and antimetastatic activity of calcitriol or its analogs in various breast cancer models was also presented [26-28]. However, recent meta-analysis has shown that the significant protective effect of high serum 25-OH vitamin D can be detected only in premenopausal patients [29]. Moreover, the results of randomized, placebo-controlled trials that included a large number of participants and lasted for 5 years have shown that vitamin D supplementation (2000 IU) has no effect on invasive cancer (including breast cancer) incidence [30]. Moreover, authors presented pro-tumoral [31] or pro-metastatic activity of calcitriol in prostate [32] and breast cancer mouse models [14].

Considering our previous results showing pro-metastatic activity of calcitriol and its analogs in young mice and their anti-metastatic activity in aged OVX mice bearing 4T1 tumors [14,15,33], as well as altered immune response occurring in elderly [34] and the well-known immunosuppressive activity of calcitriol [16], we decided to investigate the action of vitamin D compounds on the immune system during metastasis. Since the analysis of gene expression pattern in splenocytes of 4T1 tumor bearing young mice treated with calcitriol or its analogs showed increased expression of genes related to Th17 cell lineage such as: interleukin 17a (*Il17a*), interleukin 17 receptor E (*Il17re*), interleukin 1 receptor type 1 (*Il1r1*), *Il21*, RAR-related orphan receptor alpha (*Rora*), and RAR-related orphan receptor gamma (*Rorc*) [33], we focused on the role of Th17 cells population in the activity of calcitriol and its metabolite PRI-2191 in the metastasis process of 4T1 mouse mammary gland cancer in young and aged OVX mice.

## MATERIALS AND METHODS

### Splenocytes and lymph nodes used in the studies

The splenocytes and lymph nodes utilized in this study were collected from female BALB/c mice used in experiments described in our previous work [14,15]. The course of experiment on 6-8-week-old mice weighing 20-25 g was described in [14] and on 60-week-old mice weighing 24-30 g in [15] (mice obtained from the Center of Experimental Medicine of the Medical University of Bialystok, Poland). All methods used in those experiments were compliant with the EU Directive 2010/63/EU on the protection of animals used for scientific purposes and was approved by the First Local Committee for Experiments with the Use of Laboratory Animals, Wroclaw, Poland (no. of permission: 40/2014). Calcitriol (1,25(OH)_2_D_3_) and PRI-2191 (1,24(OH)_2_D_3_, tacalcitol) were obtained from the Pharmaceutical Research Institute, Warsaw, Poland. PRI-2191 was described as compound with lowered calcemic activity and increased anticancer activity as compared to calcitriol [35,36].

Briefly, 1×10^4^ 4T1 mammary gland cancer cells (American Type Culture Collection, Rockville, MD, USA) per mouse were injected orthotopically into young mice [14] - as a premenopausal model or into old mice four weeks after they had been ovariectomized [15] - as a postmenopausal model. Starting from day 7 after cells inoculation, mice were subcutaneously (s.c.) injected with 80% propylene glycol (vehicle), calcitriol (0.5 µg/kg) and PRI-2191 (1.0 µg/kg) three times a week. On days 0, 7, 14, 21, 28, and 33 after transplantation, spleens and lymph nodes were harvested after euthanasia of mice.

The experiment on young mice was repeated twice, whereas the experiment on aged OVX mice was conducted once.

### Ex vivo polarization of the immune response towards the iTh17 lymphocyte population

#### Spleen tissue preparation

Spleen samples from young and aged OVX mice (from day 21 of the experiment) were collected using sterile surgical instruments to the RPMI-1640 medium with HEPES (Gibco, Scotland, United Kingdom), 2% FBS (Gibco, Scotland, United Kingdom) and antibiotics (100 U/mL penicillin and 100 μg/mL streptomycin; Sigma-Aldrich, Saint Louis, Missouri, USA). The single-cell suspension of splenocytes was prepared by passing through sterile nylon filters (70 μm) on a petri dish and then centrifuged twice at 192×g at 4°C). For freezing in liquid nitrogen spleen cells were preserved in freezing medium containing RPMI-1640 medium (Sigma-Aldrich, Saint Louis, Missouri, USA) with 50% FBS, 10% DMSO, antibiotics and 1% 2-mercaptoethanol (Sigma-Aldrich, Saint Louis, Missouri, USA).

#### Magnetic separation of TCD4^+^ lymphocytes

After thawing and centrifuging (300×g, 4°C) spleen samples were incubated with DNase I (0.1 mg/mL) in PBS with MgCl_2_ for 15 min. Next, samples were passed through 70 μm sterile nylon filters (Corning, NY, USA) and centrifuged again (300×g, 4°C). After counting in a Bürker type hematological chamber in a 0.1% Trypan Blue solution (Sigma-Aldrich, Saint Louis, Missouri, USA) splenocytes were resuspended in 300 μl PBS with 2% FBS and 1 mM EDTA.

The separation procedure was performed using the EasySep ™ Mouse CD4 Positive Selection Kit II and the EasySep™ Magnet (STEMCELL Technologies Germany GmbH, Cologne, Germany) according to the manufacturer’s instructions. After the separation, the obtained CD4^+^ cell population was assessed for purity using flow cytometry. Therefore, the suspension of 1×10^6^ cells/mL was stained with an anti-mouse CD4-PE-Cy7 antibody (BD Pharmingen, USA) for 30 min at 4°C and then the analysis was performed using a LSR FACS Fortessa flow cytometer (BD Biosciences, San Jose, California, USA) in the FACSDiva program. In order to differentiate live cells, the DAPI dye was used.

#### Stimulation for differentiation into Th17 lymphocytes

Four hours before the start of the stimulation procedure the 96-well round bottom plates were incubated with the 10 μg/mL of anti-CD3 antibody in sterile PBS (BD Biosciences, San Jose, California, USA) at 37°C. After incubation, the plates were washed twice with sterile PBS and then culture medium was added containing the following factors: 1.5 µg/mL of anti-CD28 (Th0 conditions; BD Biosciences, San Jose, California, USA) or 1.5 µg/mL anti-CD28, 20 ng/mL of IL-6 (Biolegend, San Diego, California, USA) and 5 ng/mL of TGF-β (Th17 conditions; R&D Systems, Inc., Minneapolis, USA). Afterwards, the isolated CD4^+^ cells (0.1×10^6^ per well) were seeded at a 1:1 ratio to the wells containing the conditioned media. The cells were incubated for 4 days. After this time, stimulated cells were transferred to the 24-well plates and incubated for 4 hours at 37°C with the 50 ng/mL of phorbol myristate acetate (PMA: Sigma-Aldrich, Saint Louis, Missouri, USA) and 1μM ionomycin (Sigma-Aldrich, Saint Louis, Missouri, USA). Supernatants from splenocytes culture after centrifugation (2000×g, 4°C) were collected and the cell pellets were treated according to the RNA isolation procedure.

### Expression of genes associated with Th17 lymphocytes by real-time PCR

#### Isolation of RNA and cDNA synthesis from unstimulated splenocytes and lymph nodes samples

The total RNA was extracted using TRI reagent (Sigma-Aldrich, Saint Louis, Missouri, USA) as recommended by the manufacturer. NanoDrop 2000 (260 nm) was used to quantify and assess purity of RNA (Thermo Fisher Scientific, Waltham, MA). RNA was then verified by agarose electrophoresis.

Spleen samples from young and old OVX mice prior to RNA extraction were centrifuged using a density gradient in Ficoll Paque Premium 1.084 (GE Healthcare, Chicago, IL, USA). After thawing, the splenocytes suspension was centrifuged in a PBS solution supplemented with 2% FBS (192×g, 4°C). The cell pellet was then resuspended and layered on Ficoll, followed by centrifugation (100×g, RT).

Before reverse transcription, RNA from spleen and lymph nodes (from day 14 or 21 as an early phase of tumor progression and 28 as a late phase of tumor progression) was purified by DNAse digestion in the presence of RNase inhibitor (DNase I, RNase-free, Thermo Scientific, Waltham, Massachusetts, USA), using Veriti 9902 thermocycler (Thermo Scientific, Massachusetts, USA). In the next step, a reverse transcription reaction was performed using iScript cDNA Synthesis Kit (Bio-Rad Laboratories, Hercules, California, USA) and the following program: 5 min 25ºC, 30 min 42ºC, 5 min 85ºC and 4ºC. cDNA was stored at -20°C for further analysis. The lymph nodes from aged OVX mice did not give sufficient amount and quality of material to perform analyses.

#### Isolation of RNA and cDNA synthesis from stimulated iTh17 splenocytes samples

RNA isolation was performed immediately after splenocytes culture using the PureLink RNA Mini Kit (Thermo Fisher Scientific, Waltham, Massachusetts, USA). RNA concentration measurements and purification from genomic DNA were performed as described above. Reverse transcription reaction was carried out using the SuperScript IV VILO Master Mix with ezDNase Enzyme Kit (Thermo Scientific, Waltham, Massachusetts, USA) and the following program: 10 min 25ºC, 10 min 50ºC, 5 min 85ºC and 4ºC. cDNA samples were stored at -20ºC for further analysis.

Before proceeding with the real-time PCR, a preamplification reaction was performed using TaqMan PreAmp Master Mix (Thermo Scientific, Waltham, Massachusetts, USA) according to the manufacturer's instructions. The TaqMan probes were diluted 10× in 1×Tris-EDTA buffer (TE) to the concentration of 0.2× per reaction. The preamplification program was as follows: 10 min 95°C, 10 cycles: 15 sec. 95°C, 4 min 60°C.

#### Real-time PCR

50 ng of cDNA from untreated splenocytes, 30 ng from lymph nodes and diluted 1:5 cDNA from previously preamplified stimulated iTh17 splenocytes were used for a single real-time PCR reaction. Each sample was performed in a triplicate in a single experiment. The real-time PCR reaction was carried out in the Viia 7 device equipped with Viia 7 Software v1.1. PCR amplification cycles were performed at 2 min 50ºC, 10 min 95ºC, and then 50 cycles: 95ºC 10 sec, 58ºC 45 sec, using TaqMan probes and primers specific for following genes: *Il17a* (Mm00439618_m1), *Il17re* (Mm01189488_m1), *Il1r1* (Mn01226962_m1), *Il21* (Mm00517640_m1), *Rora* (Mm01173766_m1), *Rorc* (Mm01261022_m1), *Spp1* (Mm00436767_m1), *Vdr* (Mm00437297_m1) with TaqMan chemistry (all from Thermo Fisher Scientific, Waltham, MA). Fold-change (RQ) of target cDNA was determined using the ΔΔCt method in reference to the beta-2 microglobulin control gene (*B2m*; Mm00437762_m1) in the DataAssistTM v3.0.1 software (freeware by Applied Biosystems). *B2m* control gene was selected on the basis of our previous studies [33]. For stimulated iTh17 splenocytes the fold-change values were presented as a relative to the values obtained for non-stimulated splenocytes from control mice.

### Analysis of IL-17A production

To evaluate the secretion of IL-17A to the cell culture medium by the *in vitro* stimulated CD4^+^ lymphocytes obtained from spleens of young and aged mice, ELISA kit detecting the presence of IL-17A (Elabscience, Wuhan, China) was used according to the procedure provided by the manufacturer.

### Evaluation of the 17β-estradiol level

To estimate the 17β-estradiol level in aged OVX mice, blood specimens were collected and centrifuged (2000 x g, 15 min, 4°C). After this, plasma was collected and transferred to clean tubes and an ELISA assay was performed according to the manufacturer’s instructions (Demeditec Diagnostics GmbH, Germany). The 17β-estradiol plasma level in young mice was published previously [14].

### Flow Cytometry analysis

The single-cell suspension of spleen cells from young and aged mice (1×10^6^) resuspended in PBS with 2% FBS (GE Healthcare, Chicago, IL, USA) was incubated with CD16/CD32 antibody to block the Fc receptors. After incubation, splenocytes were stained with the anti-mouse conjugated antibodies as follows: rat CD8a-BV510, rat CD4-PE-Cy7, rat CD19-PE, hamster CD3e-APC, rat CD25-BV421, and rat CD335(NKp46)-FITC (BD Biosciences, Franklin Lakes, NJ, USA). Prior to the analysis, the cells were washed with PBS containing 2% FBS (192×g at 4°C). For data analysis, a BD LSR Fortessa cytometer with FACSDiva V8.0.1 software (BD Biosciences, Franklin Lakes, NJ, USA) was used.

### Statistical evaluation

Statistical analysis of the results was performed in the GraphPad Prism 7.03 program (GraphPad Software Inc., California, USA). The assumptions of analysis of variance (ANOVA) were tested via Shapiro-Wilk’s normality test and D'Agostino-Pearson test. Specific tests used for data analysis are indicated in the figure legends. Differences between the designated groups were marked as statistically significant when the *p* value <0.05.

**Figure 1.**
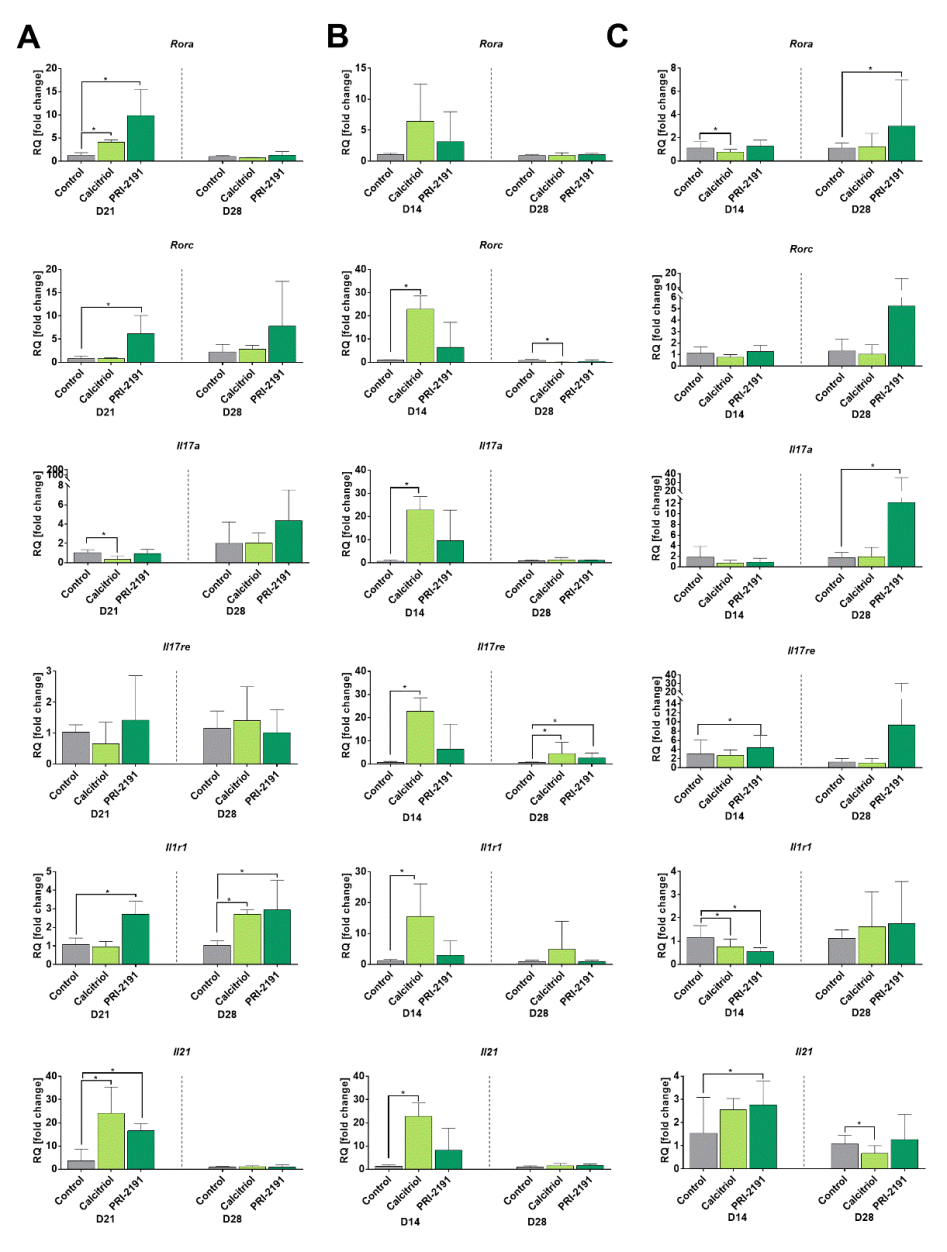
The expression of selected Th17 genes in splenocytes of young (A) and aged (B) mice and in lymph nodes of young mice (C) bearing 4T1 mammary gland tumors. 50 ng of cDNA from untreated splenocytes and 30 ng from lymph nodes were used for a single real-time PCR reaction. Each sample was performed in a triplicate in a single experiment. The real-time PCR reaction was carried out in the Viia 7 device equipped with Viia 7 Software v1.1 software. PCR amplification cycles were performed at 2 min 50ºC, 10 min 95ºC, and then 50 cycles: 95ºC 10 sec, 58ºC 45 sec using specific primers with TaqMan chemistry. Fold-change (RQ) of target cDNA was determined using the ΔΔCt method in reference to the beta-2 microglobulin control gene (B2m; Mm00437762_m1) in the DataAssistTM v3.0.1 software. Number of mice: 5-6/group. Data presented as mean with SD. Statistical analysis: Dunn’s multiple comparison test, *p<0.05.

**Figure 2.**
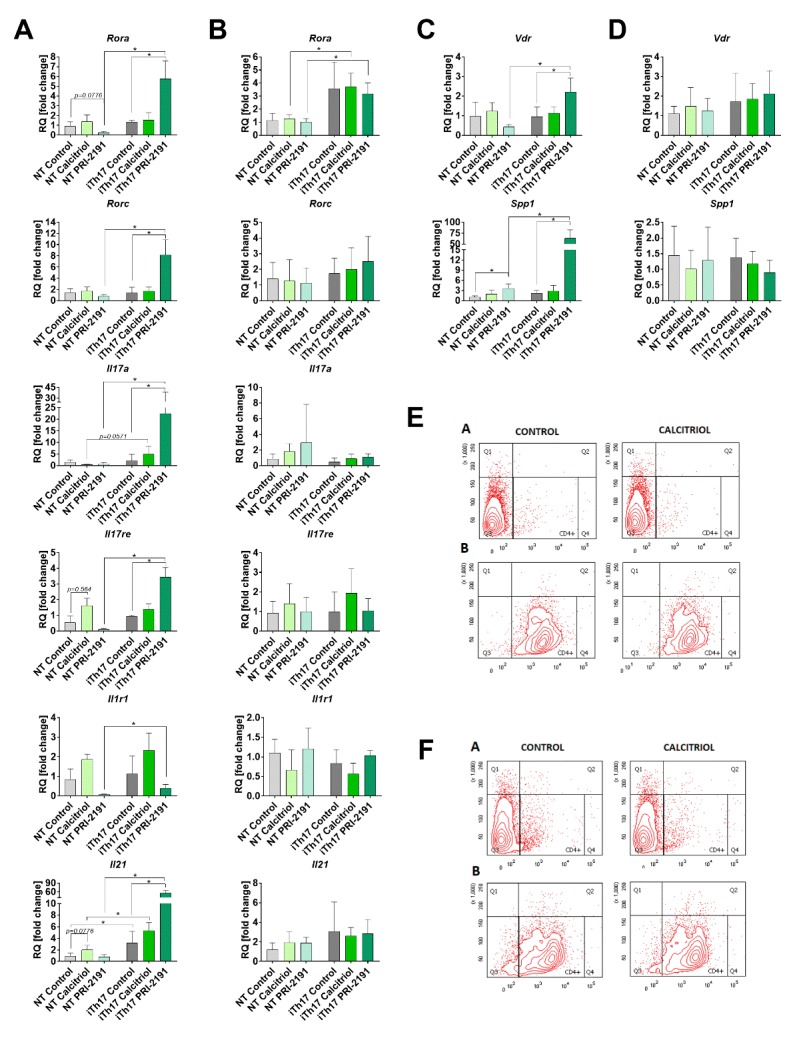
Characteristics of CD4+ splenocytes (NT) stimulated with IL-6 and TGF-β (iTh17). The expression of genes typical for Th17 cells in cells from young (A) and aged (B) mice (day 21). Expression of *Vdr* and *Spp1* in iTh17 cells induced from CD4+ splenocytes from young (C) and aged OVX (D) mice. The representative diagrams illustrating the purity of isolated CD4+ splenocytes from young (E) and aged OVX (F) control mice and mice treated with calcitriol. Panel A represents non-stained cells, panel B shows cells stained with anti-CD4 antibody. For a single real-time PCR reaction diluted 1:5 cDNA from previously preamplified stimulated iTh17 splenocytes was used. Each sample was performed in triplicate in a single experiment. Fold-change (RQ) of target cDNA was determined using the ΔΔCt method in reference to the beta-2 microglobulin control gene (*B2m*; Mm00437762_m1) in the DataAssistTM v3.0.1 software and then calculated as a relative to the values obtained for non-stimulated splenocytes from control mice. Number of mice: 4/group with the exception of control iTh17 cells from aged mice N=3. Data presented as mean with SD. Statistical analysis: Dunn’s multiple comparison test, *p<0.05.

## RESULTS

### Diversified expression of genes typical for Th17 cells in splenocytes from young and old OVX mice bearing 4T1 breast cancer and treated with calcitriol or PRI-2191

Similar expression pattern of *Rora* and *Il21* in the early (day 14 or 21) and late (day 28) stage of tumor progression was observed in young and aged OVX mice, namely the increase of its expression in mice treated with calcitriol and its analog as compared to control mice in the early stage of tumor progression and then no effect (Figure 1A and B). However, the expression of the remaining genes varied depending on the age of the mice, in particular in the late stage of tumor progression. *Rorc* increased in the early stage, was still increased on day 28 in young mice treated with PRI-2191, but in old OVX mice its expression was elevated by calcitriol in the early stage and then decreased (Fig. 1A and B). *Il17a* and *Il17re* have similar pattern of expression: calcitriol decreased the expression in young mice in the early phase of tumor progression, whereas in old OVX mice the expression of both genes increased. Next, on day 28 *Il17a* was increased by PRI-2191 in young mice, whereas no change in its expression was observed in old mice. On day 28 *Il17re* was increased in both young and old OVX mice treated with calcitriol and PRI-2191 (Fig. 1A and B). *Il1r1* was increased significantly in the early stage of tumor progression by PRI-2191 in young and by calcitriol in old mice, whereas in the late stage of tumor growth the level of its expression was increased by calcitriol and PRI-2191 only in young mice (Fig. 1A and B).

Figure 1C shows the expression pattern of the same genes in lymph nodes of young mice. *Rora* and *Rorc* have a tendency to increase on day 28 of tumor progression in mice treated with PRI-2191. *Il17a* was only increased in mice treated with PRI-2191 on day 28. *Il17re* and *Il21* were significantly increased on day 21 by PRI-2191. *Il1r1* was decreased by calcitriol and its analog on day 21 (Fig. 1C).

**Figure 3.**
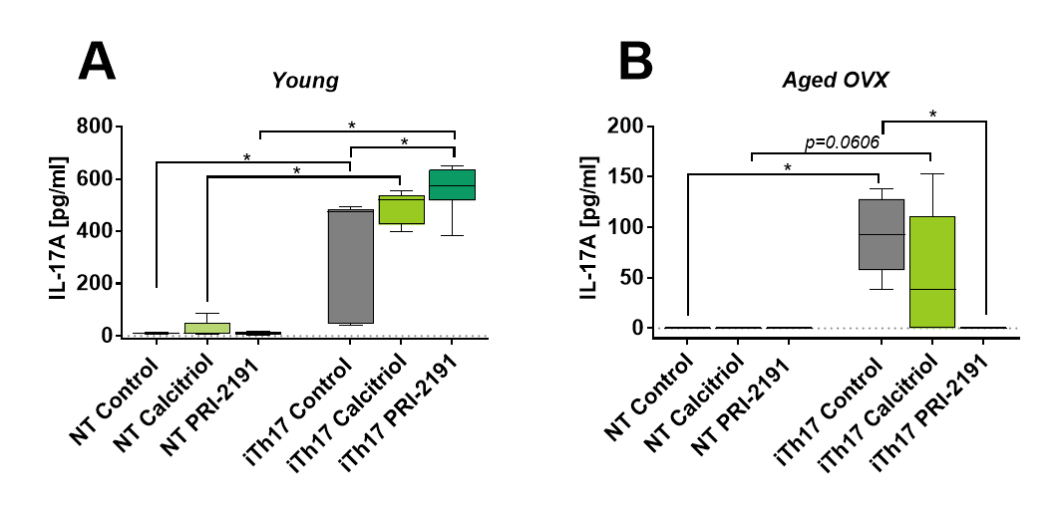
IL-17A production by iTh17 cells differentiated from CD4+ T lymphocytes (NT) from spleens of young and aged mice bearing 4T1 mammary gland cancer. Number of mice: 5-6/group. Data presented as mean with SD and min-max values. Statistical analysis: Dunn’s multiple comparison test, *p<0.05

### PRI-2191 increased the Th17 genes expression in iTh17 splenocytes from young mice bearing 4T1 mouse mammary gland cancer

CD4+ splenocytes stimulated by IL-6 and TGF-β to Th17 cells had different expression level of selected genes when harvested from young and old OVX mice. The induced iTh17 cells from splenocytes of young mice treated with PRI-2191 expressed significantly higher levels of *Il17a* mRNA as compared to iTh17 splenocytes from control tumor bearing mice (Fig. 2A). The expression of transcription factors *Rora* and *Rorc* and IL-17 receptor (*Il17re*) as well as IL-21 (*Il21*) mRNA was higher in iTh17 cells from mice treated with PRI-2191 (Fig. 2A). Vitamin D receptor mRNA (*Vdr*) as well as osteopontin (*Spp1*) significantly increased in iTh17 cells from young mice treated with PRI-2191 (Fig. 2C). Calcitriol did not affect in statistically significant manner the expression of genes in young mice (Fig. 2A). Aged OVX mice did not reveal any significant changes in the expression levels of analyzed genes (Fig. 2B). The purity of isolated CD4+ splenocytes from young (Fig. 2E) and aged OVX (Fig. 2F) were controlled by flow cytometry after staining of isolated cells with anti-CD4 antibody.

### PRI-2191 increased the IL-17A secretion by iTh17 cells from young mice but had the opposite effect in aged OVX mice

Figure 3 shows the increase in IL-17A secretion to the culture medium by iTh17 cells from young mice treated with PRI-2191 (563 pg/ml) as compared to control mice (*p*<0.05). Calcitriol also increased its level (from 307 pg/ml in control mice to 489 pg/ml in calcitriol treated mice), but this result was not statistically significant. In culture medium from iTh17 cells harvested from aged OVX mice treated with calcitriol (not significant) or PRI-2191 (*p*<0.05) the level of IL-17A was lower as compared to control tumor-bearing mice.

### Transient effect of calcitriol and PRI-2191 on 17β-estradiol plasma level in young and aged OVX mice

**Figure 4.**
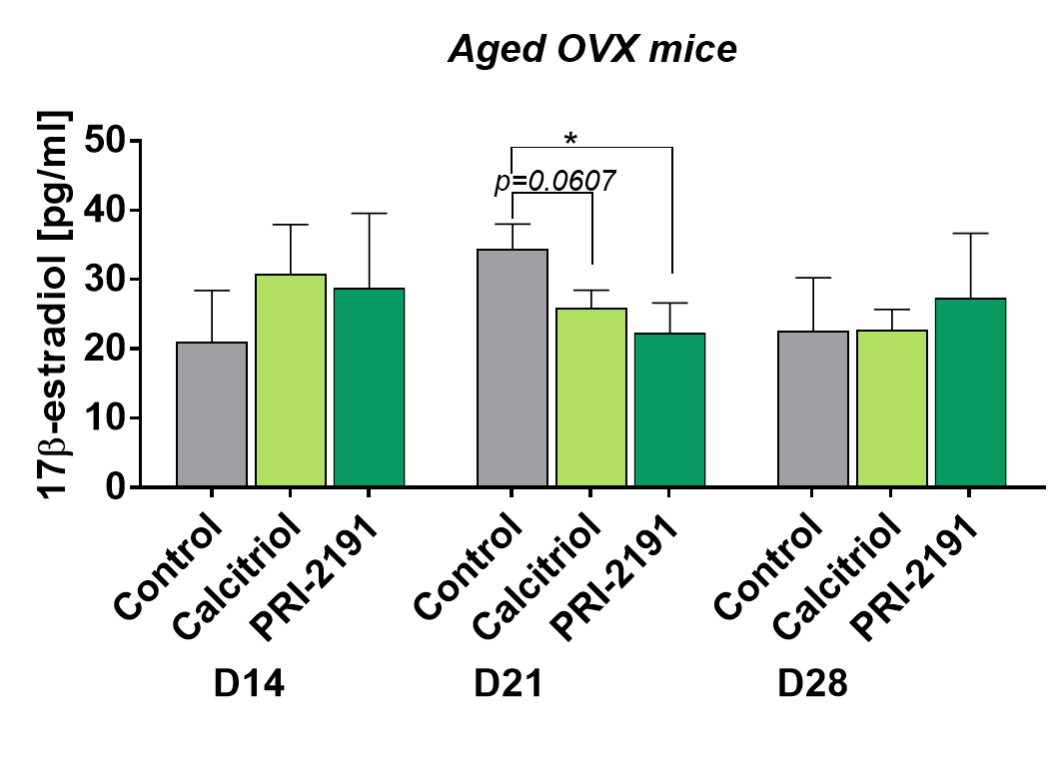
Plasma level of 17β-estradiol in aged OVX mice bearing 4T1 tumors and treated with calcitriol or PRI-2191. Number of mice: 4-5/group. Data presented as mean with SD. Statistical analysis: Dunn’s multiple comparison test, *p<0.05.

Increased 17β-estradiol level after treatment with calcitriol with similar tendency (not statistically significant) shown for PRI-2191 on day 21 was presented in our previous work in young mice [14]. Here we presented an opposite effect of calcitriol and its analog: temporary decreased 17β-estradiol in aged OVX mice plasma after treatment on day 21 (Fig. 4). In both young [14] and aged OVX mice the treatment with calcitriol or its analogs did not have any effect on day 28. The level of 17β-estradiol in aged OVX mice was measured also on day 14 and calcitriol (*p*=0.1191) and PRI-2191 (*p*=0.1033) did not affect its level significantly.

## DISCUSSION

The inflammatory response plays an important role in the development and metastasis of breast cancer and also specifically of 4T1 mouse mammary gland tumor cells [37]. In the peripheral blood, spleen and tumor tissue of mice bearing 4T1 tumors there is an increased percentage of pro-inflammatory Th17 cells that release a series of inflammatory cytokines [38]. Interestingly, the IL-17 receptor silencing results in increased apoptosis and 4T1 tumor growth inhibition *in vivo* [39]. In our studies, in which calcitriol and its analogs accelerated metastatic potential of 4T1 tumor [14], the screening of mRNA expression has shown increased expression of some genes associated with Th17 cells in the spleens of young mice [33]. We have observed that the same scheme of treatment of old OVX mice bearing 4T1 tumors leads to temporal decrease of lung metastases [15]. The differences in metastatic process between young and aged OVX mice in our previous studies was the most visible on the 28 day of observation. In young mice calcitriol increased the number of lung metastatic foci in 27% and PRI-2191 in 127% [14]. In aged OVX mice 29 and 70% decrease of lung metastases was observed after treatment with calcitriol and PRI-2191, respectively [15]. At the end of observation stimulation of metastases (confirmed by histopathological examination) was still observed in young mice [14], whereas antimetastatic effect transitorily observed on day 28 in aged OVX mice, on day 33 disappeared [15]. Therefore, in the present work we analyzed the expression of several genes related to the Th17 cells in tissues collected from young and old OVX mice bearing 4T1 tumors and treated with vitamin D compounds. In splenocytes of young mice, the expression of some of them increased in those treated with calcitriol or PRI-2191. They include the *Rora* (encoding RORα) transcription factor and *Il21* gene. IL-21 is produced by Th17 cells and stimulates their formation on the autocrine route both in mice and humans [40,41]. In addition, PRI-2191 increased the expression of the *Rorc* (encoding RORγt*)* transcription factor and the *Il1r1* gene - the another promoter of Th17 cell differentiation and regulator of RORγt expression [42]. RORγt and RORα can synergistically induce the differentiation of mouse and human Th17 cells [43,44]. On day 28, both compounds increased *Il1r1* mRNA level. Moreover, on day 28 calcitriol analog stimulated the expression of *Rorc* and *Il17a* genes. In the spleens of aged OVX mice, the effect of tested compounds on the expression of genes associated with Th17 cells in the advanced stage of the disease was not as significant as in the case of young mice, with the exception of *Il17re* gene. Interestingly, the expression of *Rora* was diminished in aged OVX mice treated with calcitriol. However, in the early stage of cancer progression, calcitriol significantly increased the expression of almost all tested genes. This effect was higher than that observed for PRI-2191, unlike in young mice where the activity of PRI-2191 was clearer.

**Figure 5.**
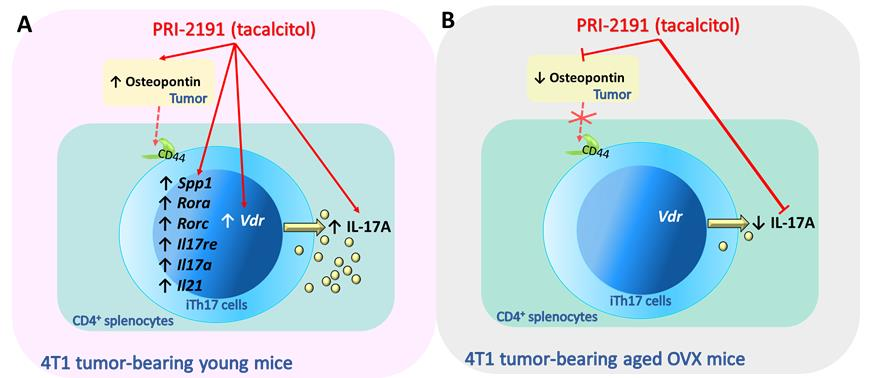
Summary of the effects on iTh17 cells of PRI-2191 (tacalcitol) treatment of young and aged OVX mice bearing 4T1 mammary gland cancer. (A) PRI-2191, by increasing osteopontin level, may indirectly potentiate the differentiation of Th17 cells and the secretion of IL-17 by them. The direct effect of PRI-2191 after binding with VDR on the expression of osteopontin and genes responsible for Th17 differentiation is also considered. (B) In old OVX mice, the observed reduction in the secretion of osteopontin may contribute to reduced secretion of IL-17. Continuous red lines show the effects of PRI-2191 observed in the orthotopic 4T1 mouse breast cancer model, where PRI-2191 stimulated metastasis in young mice and inhibited in old OVX mice. Dotted lines indicate the effects of osteopontin on Th17 cells differentiation (through CD44 or other receptors) observed by other authors in other models (non-cancerous).

The analyses performed on unstimulated splenocytes have shown some differences between young and old OVX mice, which are particularly enhanced in a more advanced phase of tumor progression. For detailed analysis of Th17 response, we stimulated CD4^+^ splenocytes harvested from young and aged OVX mice bearing 4T1 tumors treated with calcitriol and its analog, with TGF-β and IL-6 to induce Th17 cells (iTh17) *ex vivo*. This study revealed that iTh17 cells from young mice treated with PRI-2191 released higher IL-17A levels than control, not-treated mice and a reverse effect (decreased IL-17A secretion) was observed in old OVX mice. This effect correlated with the *Il17a* mRNA expression. IL-17 is a proinflammatory cytokine with proven pleiotropic effect. Studies show that the pro-tumor effect of IL-17 supporting tumor angiogenesis increases in the chronic phase of cancer and inflammation development, and dominates over anti-cancer effects, promoting the expansion of cytotoxic T lymphocytes and other immune cells fighting cancer [45,46]. Stimulating effect on angiogenesis was also reported in 4T1 tumor model after treating mice with recombinant IL-17A [12]. In the 4T1 mouse model (young mice), in which PRI-2191, and less clearly also calcitriol increased iTh17 IL-17A secretion, we also observed an increase in blood perfusion within the tumor [14]. However, in aged OVX mice we did not observe any effect of the treatment on tumor blood perfusion [15].

Studies indicate that Th17 cells highly express VDR. As a result, calcitriol can modulate the expression of IL-17A in both mouse and human T lymphocytes. Most studies suggest that calcitriol leads to a reduction in the recruitment of Th17 cells and IL-17 secretion via the VDR receptor-mediated pathway [47,48]. However, there are studies in which no correlation between Th17 cells and the level of circulating 25-OH vitamin D was observed, even after additional supplementation with vitamin D_3_. In the study of multiple sclerosis in patients who did not receive previous supplementation with vitamin D_3_, despite its high level, no correlation was observed between vitamin D_3_ status and individual T cell populations; similar results were observed after 12-week supplementation [49,50]. There are several potential causes for conflicting reports on this subject. It seems that among others, vitamin D_3_ status in the organism, the dose used, and the level of estradiol which is involved in *Vdr* gene expression in Th17 cells play a significant role [47]. What is more, the synergistic effects of vitamin D_3_ and estradiol have been demonstrated. It has been shown that increased estrogen synthesis is mediated by vitamin D_3_, while estrogens are essential for the VDR receptor expression and function in inflammation of the central nervous system [51]. Interestingly, our results have shown increased 17β-estadiol plasma levels after calcitriol treatment in young mice [14], whereas PRI-2191 and to a lesser extent also calcitriol decreased its plasma level in aged OVX mice bearing 4T1 tumors. However, this divergent estrogen regulation in both groups of mice (pre- *vs* post-menopausal models) is only transient, and is not directly relevant for the expected effect on Th17 cells, as it was previously reported that estradiol inhibits Th17 differentiation and IL-17 production by inhibiting RORγT expression [52]. However, induced iTh17 lymphocytes isolated from spleens of young mice showed increased expression of *Vdr* after treatment with PRI-2191, while in the aged OVX mice model such effect was not observed. A similar correlation was observed in the lymph nodes of young mice, namely PRI-2191 stimulated the expression of selected genes associated with Th17 cells (current work) in the presence of increased expression of the *Vdr* receptor [33].

In addition to the genes typical for the Th17 subpopulation, the expression of the *Spp1* gene encoding osteopontin (OPN) in the iTh17 cells was evaluated. In breast cancer patients, the high expression of OPN correlated with lymph node metastasis, and advanced tumor stage [53]. After treatment with PRI-2191 in young mice, the expression of the *Spp1* mRNA increased. According to the current state of knowledge, OPN is required in dendritic cells for induction of Th17 cells differentiation [54] and production of IL-17 [55]. In addition, studies of acute coronary syndrome have shown OPN to correlate positively with inflammation through a direct effect on IL-17 producing cells [56]. Moreover, the direct effect of OPN on the differentiation of Th17 cells is exerted through interactions with its receptors [57]. In our studies, the increased expression of *Spp1* in iTh17 cells of young mice correlates with the intensity of gene expression characteristic for these proinflammatory cells. Furthermore, in our previous studies we observed a significant increase in osteopontin tumor tissue level of young mice bearing 4T1 tumor and treated with calcitriol and its analogs [14], whereas in tumor tissue of aged OVX mice its level was significantly diminished [15]. Calcitriol directly stimulates OPN expression in various cells through vitamin D responsive elements (VDRE) in *Spp1* gene [58,59]. Therefore, its increase in 4T1 tumor tissue [14], lymph nodes [33] or iTh17 cells (current work) from young mice was predictable and may have contributed to the observed enhanced differentiation of iTh17 cells and the production of IL-17A in young mice (Fig. 5A).

The CD8^+^ lymphocytes correlate positively with the secretion of IL-17 and Th17 cell activity [60-62]. Cell phenotype analysis in the spleens of young 4T1 bearing mice revealed an increase in the percentage of cytotoxic CD8^+^ lymphocytes in response to treatment with calcitriol and its analogs on day 14, 21 and 28 of observation [33]. Stimulation of selected genes associated with Th17 lymphocytes by calcitriol and its analogs correlated with the percentage of CD8^+^ cells also in the lymph nodes of young mice [33]. A similar effect was not observed in the aged OVX mice, i.e. the applied vitamin D compounds lowered the expression of the CD8^+^ marker in the spleen (Supplementary Fig.1), which correlated with a lower secretion of IL-17 by stimulated splenocytes and changes in Th17 genes expression. What is more, the applied compounds induced immunosuppression in young mice, which was shown mainly by an increase in the percentage of Treg cells in the spleen, as well as IL-10 and in the tumor tissue, especially in the initial stages of tumor development [33]. In aged OVX mice no similar effect of the compounds was observed, namely CD25^+^ splenocytes population was reduced in mice treated with vitamin D compounds (Supplementary Fig.1).

### Conclusions

Based on the studies carried out on the 4T1 mouse model, age-dependent immunomodulatory effects of vitamin D derivatives were found. Induced iTh17 cells derived from spleens of mice treated with PRI-2191 are stimulated at the gene expression level (*Rora*, *Rorc*, *Il17re*, *Il17a*, *Il21*), and the IL-17A secretion due to increased expression of the *Vdr*. Moreover, increased expression of *Vdr*, lead to increased expression of *Spp1* what enhanced the differentiation of Th17 cells (Fig. 5A). In aged OVX mice opposite effects were observed (Fig. 5B). The explanation of the exact mechanisms responsible for the observed differences in the response of young and old OVX mice to calcitriol and PRI-2191 requires further investigation.

## Supplementary Materials

The Supplemenantry data can be found online at: www.aginganddisease.org/EN/10.14336/AD.2020.0228.
