# Supplementary material for: Divergent Effect of Tacalcitol (PRI-2191) on Th17 Cells in 4T1 Tumor Bearing Young and Old Ovariectomized Mice
Source: Aging Dis. 2020 Mar 9;11(2):241–53. doi: 10.14336/AD.2019.0618 (PMC7069462; doi:10.14336/AD.2019.0618)
Supplement: Supplementary file 1 — The Supplemenantry data can be found online at: www.aginganddisease.org/EN/10.14336/AD.2020.0228. [file AD-11-2-241-s.pdf]

SUPPLEMENTARY DATA

Divergent Effect of Tacalcitol (PRI-2191) on Th17 Cells in 4T1 Tumor Bearing Young and Old Ovariectomized Mice

Agata Pawlik[1,#], Artur Anisiewicz[1], Beata Filip-Psurska[1], Dagmara Klopotowska[1], Magdalena Maciejewska[1], Andrzej Mazur[2], Joanna Wietrzyk[1*]

[1]Department of Experimental Oncology, Hirszfeld Institute of Immunology and Experimental Therapy, Polish Academy of Sciences, Wroclaw, Poland
[2]Université Clermont Auvergne, INRA, UNH, Unité de Nutrition Humaine, F-63000 Clermont-Ferrand, France

© 2019. Pawlik A et al. Published online at http://www.aginganddisease.org/EN/10.14336/AD.2019.0618

SUPPLEMENTARY DATA

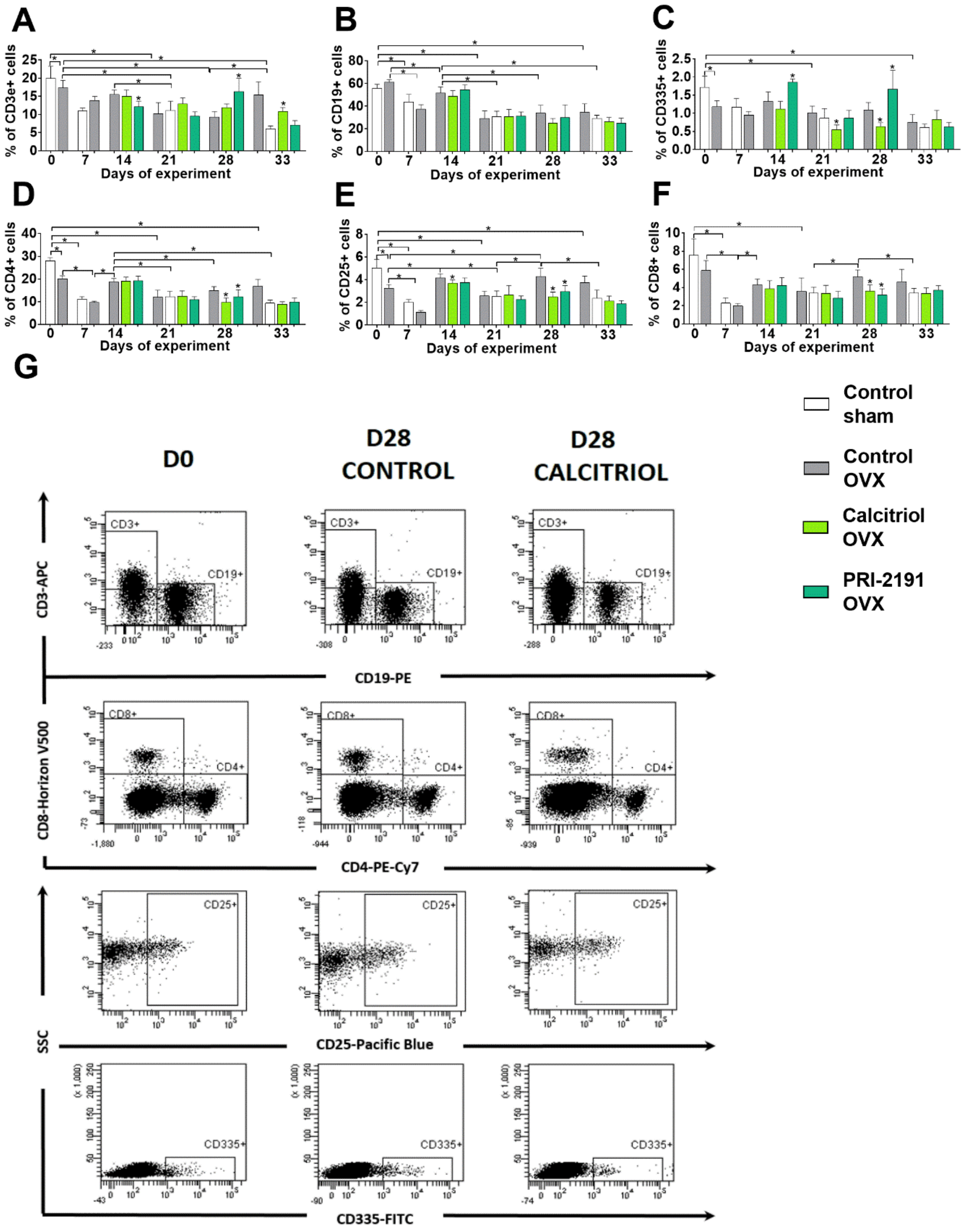

Supplementary Figure 1. **Splenocytes phenotype: aged ovariectomized (OVX) mice bearing 4T1 mouse mammary gland tumors and treated with calcitriol or PRI-2191.** The single-cell suspension of spleen cells from aged mice (1×10^6) was stained with the anti-mouse conjugated antibodies as follows: rat CD8a-BV510, rat CD4-PE-Cy7, rat CD19-PE, hamster CD3e-APC, rat CD25-BV421, and rat CD335(NKp46)-FITC. Control sham: sham-operated tumor-bearing mice; Control OVX: OVX tumor-bearing mice; D0 means day of tumor cells inoculation, 4-weeks after sham-operation or ovariectomy. Number of mice: 5-7/group with the exception of day 33: PRI-2191 and Control OVX = 3. Data presented as mean with SD. Statistical analysis: Dunn's multiple comparison test. *$p<0.05$ as compared to Control OVX at the same day of observation or as indicated.

© 2019. Pawlik A et al. Published online at http://www.aginganddisease.org/EN/10.14336/AD.2019.0618
